# Ranking Universities of Medical Sciences as Public Health Services Provider Institutions in Iran: A Result-Chain Analysis

**DOI:** 10.34172/aim.2022.37

**Published:** 2022-04-01

**Authors:** Nader Jahanmehr, Arash Rashidian, Farshad Farzadfar, Ardeshir Khosravi, Mohammad Shariati, Ali Akbari Sari, Soheila Damiri, Reza Majdzadeh

**Affiliations:** ^1^Department of Health Economics, Management and Policy, Virtual School of Medical Education & Management, Shahid Beheshti University of Medical Sciences, Tehran, Iran; ^2^Prevention of Cardiovascular Disease Research Center, Shahid Beheshti University of Medical Sciences, Tehran, Iran; ^3^Department of Health Management and Economics, School of Public Health, Tehran University of Medical Sciences, Tehran, Iran; ^4^Department of Global Health and Public Policy, School of Public Health, Tehran University of Medical Sciences, Tehran, Iran; ^5^Knowledge Utilization Research Center, Tehran University of Medical Sciences, Tehran, Iran; ^6^Non-Communicable Diseases Research Center, Tehran University of Medical Sciences, Tehran, Iran; ^7^Center for Primary Health Care Management, Ministry of Health and Medical Education, Tehran, Iran; ^8^Department of Community Medicine, School of Medicine, Tehran University of Medical Sciences, Tehran, Iran; ^9^Department of Epidemiology and Biostatistics, School of Public Health, Tehran University of Medical Sciences, Tehran, Iran

**Keywords:** Factor analysis, Healthcare disparities, Health status disparities, Public health, Primary health care

## Abstract

**Background:**

Universities of medical sciences (UMSs) in Iran have geographic catchment areas (normally a province) in which they are responsible for public health services as well as provision of care by public providers. The present study strived to analyze and rank the performance of the medical sciences universities in improving the public health and primary healthcare.

**Methods:**

Data on 41 indicators on the output (16 indicators), outcome (16 indicators), and impact (9 indicators) levels were extracted from various data sources. Principal component analysis (PCA) was used to calculate the weight for each of the indicators. The score range for each level of performance is between 0 and 1. A score of 1 indicates the highest and a score of 0 indicates the lowest level of performance. Finally, the UMSs were ranked by their scores.

**Results:**

The national mean performance scores of the UMSs on the output, outcome, impact, and the composite indicator levels were 0.756, 0.641, 0.561, and 0.563, respectively. The results show that the changes in performance scores at different levels of the results chain are remarkable.

**Conclusion:**

The national mean performance of the UMSs of Iran is not satisfactory. However, there is considerable dispersion in their performance. Designing effective interventions in proportion to the conditions of universities on different levels of the results chain, developing a robust information system, conducting continuous monitoring and evaluation of public health are recommended for balanced improvements in public health and primary healthcare indicators in the country.

## Introduction

 Due to intrinsic and instrumental characteristics, health is considered as one of the key dimensions of wellbeing and one of the primary requisites for economic growth and social solidarity.^1^ In the past decades, the key role of health in international development has been acknowledged and numerous attempts have been made to reduce the mortality and morbidity rate, universally or through a focus on specific population subgroups.^2^ As a result of these attempts, considerable progress has been made in improving health indicators on the global level.^3,4^ However, the widening gap in health achievements has become a major concern for policy-makers. There are differences in health outcomes across and within countries.^1^ Various studies, for example, have revealed evidence of the differences in health outcomes in Slovakia,^5^ the United States,^6^ Korea,^7^ Australia,^8^ China^9^ and Indonesia.^10^

 Health disparities refer to the differences in health outcomes and their determinants among different population groups defined by social, demographic, environmental, and geographical characteristics.^11^ Monitoring regional health inequalities is one of the useful actions taken to unveil the geographical differences in health and to develop equity-oriented interventions. To explain the logic of measuring regional disparities, it could be stated that the population residing in a region is under the same conditions, which can directly or indirectly affect its health. These conditions include the inputs and processes of the health system, the availability of other services (e.g. education), local infrastructure, climate, environmental pollution, and local culture. Monitoring regional health inequalities can reveal important evidence that can support targeting by health programs and policies.^12^ Evidence has revealed inter-provincial and intra-provincial disparities in health indicators in Iran, which are not acceptable.^13-17^ For instance, despite the increased life expectancy of women in all provinces of Iran, there is a greater than 8.3-year difference across Iranian provinces.^13^

 The difference in design, content, and management of health systems is one of the reasons for the differences in health outcomes.^18^ Hence, improving the performance of health systems is a priority in the discussions among governments, policy-makers, and healthcare providers around the globe.^19^ Decision-makers on all levels need to quantify the differences in the health system performance, identify the determining factors, and formulate policies that can improve the results in a variety of settings.^18^ Furthermore, achieving balanced regional development and reducing non-homogeneity and regional disparities are contingent on the understanding and analysis of the characteristics of each region regarding its position in the entire system.^20^ Comparable information on the performance of the health system and the principal factors determining the difference in the performances of different systems can also result in scientific health policies at the national and regional levels.^18^

 One of the ultimate means of reducing the health disparities is increasing the quantity and quality of primary health care (PHC) services for the people who are vulnerable due to the effects of a set of health determinants.^21^ PHC is the first and the most important point of contact between the health system and the public whereby essential, continuous, comprehensive, and coordinated care is provided. These health care services increase the accessibility and quality of care and improve the health outcomes and equity of health outcomes.^22^ Hence, countries have started developing this sector as an essential element of their economic and social growth.^23^

 In Iran, considerable changes were made in the organizational structure of the health system in 1985 including the establishment of independent regional units, known as Universities of Medical Sciences (UMSs).^24^ These universities have geographic catchment areas (normally a province) in which they are responsible for public health services as well as provision of care by public providers. Currently, Iran has 63 UMSs and faculties that implement the macro-policies and plans formulated by the Ministry of Health and Medical Education.^25^ Every UMS has a health deputy responsible for providing the first-level services including public health and primary healthcare.^26^ The public health deputies that provide health services have similar structures and hierarchies throughout the country and each of them solely provides services to a specific community. The present study strived for investigating the regional disparities in the health achievements related to the performance of the Iranian public health and primary healthcare systems using the results-chain framework. Also, UMSs were ranked based on their performances at different levels of the results chain. The health ranking presented in this study can be used as a catalyst to improve health by focusing on areas requiring improvement. When the media and community leaders are informed of the problem areas, they can approve the health policies and plans based on the provided evidence to improve the health outcomes.^27^

## Materials and Methods

 The goal of this applied cross-sectional study was to illustrate and rank the performances of 45 UMSs in Iran, regarding their role in improving public health based on the results-chain framework of their public health deputies and composite measures. To achieve this, the results chain framework proposed by Jahanmehr et al^28^ was used. A results chain framework shows how the system inputs and processes are reflected in the outputs, outcomes, and impacts. Hence, it can demonstrate the performance of health systems interventions.^29^ The results chain used in this study was composed of three levels: the output level which focuses on the health services coverage (16 indicators); the outcome level which focuses on health behavior and risk factors (16 indicators); and the impact level which focuses on the mortality and communicable and non-communicable diseases (NCDs) (9 indicators). The performances of universities were evaluated and ranked based on the 2010 data on four levels: output, outcome, impact, and a composite index (i.e. the combination of the three mentioned indices). Data was extracted from various sources includes: the census statistics published by the Iran Statistics Center, the statistical reports by the public health deputy of the Ministry of Health and Medical Education, the vital horoscope indicators, the results of the Multiple Indicator Health and Demographic Survey (IrMIDHS)^30^ and the results of the national survey of the risk factors of NCDs.^31^ Details about the selected indicators and the specific source of each of them and their descriptive statistics are presented in Table S1 and S2 in Supplementary file 1. Factor analyses were carried out in STATA 12 to assign weights of indicators. After preparing and assessing data quality, assigning weights to the indicators and ranking of the universities performances were done in the following steps.

###  Process and Criteria for Selecting the Indicators

 By taking the opinion of experts as well as studying the available scientific resources and reviewing the experience of other countries, in meetings held at different times by the research team, after discussing the goals and strategies of the public health deputy of the Ministry of Health and Medical Education and information needs of various stakeholders, areas and indicators for evaluating the performance of public health deputies were selected. Also, the scientific criteria for selecting the indicators in this study were:

- Covering the areas of activity of the public health deputy
- Relevance of indicators
- Availability of information at the university level
- Measurability of indicators
- Updates of indicators at different times by reliable sources

###  Investigating the Basic Assumptions of Analyzing the Fundamental Hypotheses for the Factor Analysis

 The Kaiser-Meyer-Olkin (KMO) test was carried out to assess the sampling adequacy to obtain reliable results. The result of this test varies between 0 and 1, with a KMO result higher than 0.5 showing adequacy of data correlation for the factor analysis. In this study, the KMO result was higher than 0.5 at all levels. Therefore, the Bartlett Test of Sphericity was carried out to determine the adequacy of data. If the hypothesis on the lack of correlation among the variables is rejected, the data will be suitable for factor analysis. In all of the factor analyses in this study, the result of Bartlett’s test was significant.

###  Principal Component Analysis 

 The weight of the indicators is not the same in measuring the performance of public health deputies, so we used the factor analysis method to measure the weight for each of the indicators. Principal component analysis (PCA) is a practical method for determining the internal weights of a set of indicators to create a composite index. The purpose of this weighting method is to describe the indicators with a set of the most important vertical rotation factors.

###  Determining the Number of the Primary Factors at Different Performance Evaluation Levels

 In terms of the constructing composite indicators handbook,^32^ three criteria were selected, namely the eigenvalue > 1, the over 10% explanation of the total variance of the dataset by each selected factor, and the over 60% explanation of the total variance of the dataset by the set of factors, as well as the scree plot that was used to select the factors.

###  Calculating the Indicators’ Weights

 The important factors were extracted using the criteria mentioned earlier in the previous step. After extracting the factor loadings of all indicators, varimax rotation of all factors was carried out and the rotated factor loadings were obtained. The factor loadings of all indicators were raised up to the power of two, and the largest result was selected as the weight of the area from the factors for all indicators. The weight calculated for each indicator was multiplied by the variance ratio described by the related factor, and the result was used as the weight of that indicator. To determine the share (%) of each indicator, the indicator weight was converted to the share of one scale (% of 1) and the result was selected as the final weight of that indicator. The different steps of assigning weights to the output, outcome and impact indicators are presented in Tables S3 to S6 in Supplementary file 1. To rank the performances, the weighted sum of each of the three levels, namely the output, outcome, and impact levels, was calculated through a separate factor analysis by repeating the aforesaid steps. The only difference was in the promax rotation at this level. Hence, the final composite index calculated in this study was the result of 41 indicators at different levels of the results chain, which presented a comprehensive image of the performance of universities regarding their role in improving health in different regions of Iran. A composite index is the result of the mathematical combination of a set of indicators into a single number. This index can describe an entire set of indicators.^33^ Accordingly, some of the potential applications of the composite indices include comparing the health of a population to other populations, monitoring the variations of health in a given population, and identifying and quantifying the public health disparities in a population.^34^

###  The Score and Ranking Formula

 After assigning weights to all indicators values, the performance score for each university was formed based on the following formula. The score is stated as a decimal.


$$\begin{array}{l} Performance\;score\;of\;each\;university=\\ \frac{Inidicator\;value\;of\;each\;university-national\;mean}{S\tan dard\;deviation\;of\;all\;university\;value} \end{array}$$

Often referred to as a “Z-score”, this score indicates the number of standard deviations a university is above or below the national mean. Universities that have a higher value than the national average will have a positive performance while those with a lower value will have a negative performance. Scores are calculated to three decimal places and, in all performance levels, the highest score that each university can receive is equal to 1 and the lowest score is equal to zero. Where a value for the country overall is not available, the national mean is set at the average value of the universities in each level of result chain performance. Ranking is the ordering of universities according to their performance scores.

###  Adjustment of Direction in the Indicators

 Before performing the analysis, all indicators included in the study were adjusted in the same direction (same sign) in terms of the impact on the result. For this purpose, the indicators that had the nature of a negative effect were converted into positive indicators by making an adjustment through subtracting all observations from the largest observation. By doing this, when interpreting the indicators, an increase in all of them is considered desirable.

## Results

 The present research was an attempt to evaluate and rank the performances of the UMSs of Iran in improving the public health conditions based on the results-chain of the public health deputies of these universities. Using the chain framework, the performances of the universities were evaluated and ranked at four levels including the output, the outcome, the impact, and the composite index levels. The factor analysis method was used to assign weights to the indicators at each of the four above-mentioned levels. Table 1 shows the weights assigned to each indicator. As seen, in creating the composite index, the highest weight was related to the outcome indicators.

**Table 1:** Final Weights of the Indicators at the Outcome, Output, Impact, and Composite Indicator Levels

**Output Indicators**	**Final Weight**	**Outcome Indicators**	**Final Weight**
Neonates weighed at the time of birth	0.165	Prevalence of hypertension	0.021
Percentage of microbiologic quality of drinking water in rural areas	0.067	Prevalence of obesity	0.108
Percentage of microbiologic quality of drinking water in urban areas	0.063	Prevalence of overweight or obesity	0.147
Accessibility of clean drinking water in rural areas	0.014	Prevalence of hypothyroidism in screened neonates	0.016
The percentage of consumers of optimal drinking water resources.	0.126	The rate accurate awareness of HIV prevention in women aged between 15 and 54 years old	0.018
Sanitary disposal of children’s feces	0.016	The percentage of children who are exposed to the smoke of cigarettes at least once a week.	0.062
Percentage of refined iodized salts in public places and food stores	0.008	The percentage of children below the age of 5 suffering from diarrhea	0.074
The percentage of workshops covered by professional health services	0.053	Prevalence of extreme slimness in children below the age of 5	0.044
Percentage of employees covered by occupational examinations	0.011	Prevalence of severe dwarfism in children below the age of 5 years	0.131
Prenatal care coverage (at least two times)	0.029	The rate of exclusive breastfeeding up to the age of 6 years	0.041
Labors carried out in health centers (the public and private sectors)	0.143	The prevalence of low birth weight	
Prenatal care provided by educated or trained caregivers	0.053	The percentage of smokers who smoke daily - men	0.018
The ratio of women who have given birth and have received prenatal supplements.	0.025	The prevalence of the low intake of fruit and vegetable	0.039
The coverage of users of contraception devices	0.108	The prevalence of under-activity	0.047
Children aged between 12 and 23 months, who are vaccinated against measles.	0.094	Successful treatment of new pulmonary tuberculosis cases with positive smears	0.008
Accessibility of clean bathrooms in villages	0.037	Bottle feeding	0.065
**Impact Indicators**	**Final Weight**	**Composite Indicator Weights**	**Final Weight**
Death of neonates below the age of 1 month in every 1000 live births	0.178	Output indicators score	0.334
Death of children below the age of 5 years in every 1000 live births	0.179	Outcome indicators score	0.437
The still birth to live birth ratio in every 1000 births	0.058	Impact indicators score	0.277
The prevalence of diabetes in villagers over the age of 30 years	0.024		
The standardized rate of incidence of women’s cancers	0.171		
The standardized rate of incidence of men’s cancer	0.148		
The total incidence of new tuberculosis cases	0.048		
Incidence of measles	0.125		
The incidence of malaria	0.065		

 At the output level, the average performance score of the universities was 0.756, while 65% of the universities gained the scores above average. Zahedan University gained the lowest score (0.009) and the 45^th^ rank, while Qazvin University gained the highest score (0.950) and the first rank among all universities (Table 2).

**Table 2:** Public Health Scores and Ranks in Regions Covered by the Universities of Medical Sciences

**Rank**	**Output- process level**		**Outcome Level**		**Impact Level**		**Composite Index Level**	
	**Medical University**	**Score**	**Medical University**	**Score**	**Medical University**	**Score**	**Medical University**	**Score**
1	Qazvin	0.950	Shahrud	0.936	Qom	1.00	Ilam	1.000
2	ChaharM & Bakhtiari	0.930	Fasa	0.881	Hormozgan	0.953	Bushehr	0.965
3	Ilam	0.928	Bushehr	0.866	Ilam	0.948	Birjand	0.863
4	Kurdistan	0.916	Gilan	0.848	Zabol	0.931	Gilan	0.840
5	Kashan	0.906	Birjand	0.837	Zanjan	0.893	Golestan	0.819
6	Neyshabur	0.905	Ilam	0.833	Bushehr	0.862	Shahrud	0.787
7	Dezful	0.892	Zabol	0.819	Zahedan	0.858	Ardabil	0.760
8	Gonabad	0.890	Hamadan	0.797	Bojnourd	0.856	ChaharM & Bakhtiari	0.756
9	Isfahan	0.889	Ardabil	0.788	ChaharM & Bakhtiari	0.848	Bojnourd	0.749
10	Golestan	0.885	Mazandaran	0.783	Birjand	0.758	Mazandaran	0.733
11	Bushehr	0.880	Bojnourd	0.779	Golestan	0.767	Azerbaijan-West	0.725
12	Gilan	0.876	Jahrom	0.774	Kohgiluyeh & BoyerA	0.729	Fasa	0.710
13	Sabzevar	0.869	Shahid-Beheshti	0.771	Ardabil	0.726	Hamadan	0.697
14	Zanjan	0.868	Shiraz	0.770	Qazvin	0.685	Lorestan	0.666
15	Markazi	0.867	Iran	0.758	Azerbaijan-West	0.697	Kermanshah	0.641
16	Semnan	0.864	Azerbaijan-Eeast	0.746	Lorestan	0.675	Gonabad	0.637
17	Torbat-Heidariye	0.840	Golestan	0.742	Gilan	0.598	Sabzevar	0.626
18	Mazandaran	0.840	Kermanshah	0.737	Babol	0.538	Shiraz	0.621
19	Azerbaijan-West	0.839	Mashhad	0.709	Mazandaran	0.532	Isfahan	0.606
20	Mashhad	0.835	Azerbaijan-West	0.709	Kermanshah	0.515	Mashhad	0.605
21	Qom	0.825	Gonabad	0.704	Hamadan	0.493	Jahrom	0.600
22	Ahvaz	0.812	Sabzevar	0.700	Kurdistan	0.484	Torbat-Heidariye	0.598
23	Tehran	0.812	Torbat-Heidariye	0.691	Jiroft	0.479	Babol	0.593
24	Birjand	0.810	Lorestan	0.687	Fasa	0.478	Zanjan	0.593
25	Yazd	0.807	Isfahan	0.686	Kerman	0.478	Kurdistan	0.585
26	Hamadan	0.788	Hormozgan	0.675	Rafsanjan	0.478	Azerbaijan-West	0.582
27	Babol	0.780	Babol	0.665	Shiraz	0.477	Hormozgan	0.572
28	Lorestan	0.778	Tehran	0.657	Jahrom	0.472	Shahid-Beheshti	0.525
29	Kermanshah	0.769	Kohgiluyeh & BoyerA	0.634	Shahrud	0.458	Kohgiluyeh & BoyerA	0.524
30	Shahrud	0.756	Zahedan	0.623	Semnan	0.448	Iran	0.519
31	Ardabil	0.753	ChaharM & Bakhtiari	0.603	Ahvaz	0.433	Neyshabur	0.516
32	Iran	0.726	Kurdistan	0.585	Dezful	0.427	Qazvin	0.504
33	Azerbaijan-Eeast	0.721	Jiroft	0.565	Azerbaijan-Eeast	0.422	Semnan	0.502
34	Shahid-Beheshti	0.718	Neyshabur	0.552	Neyshabur	0.402	Qom	0.499
35	Shiraz	0.714	Semnan	0.544	Sabzevar	0.402	Tehran	0.480
36	Fasa	0.700	Ahvaz	0.528	Torbat-Heidariye	0.400	Ahvaz	0.449
37	Jahrom	0.681	Kerman	0.489	Gonabad	0.390	Zabol	0.406
38	Rafsanjan	0.669	Zanjan	0.443	Mashhad	0.384	Kashan	0.327
39	Bojnourd	0.665	Qazvin	0.379	Isfahan	0.352	Jiroft	0.269
40	Kohgiluyeh & BoyerA	0.592	Kashan	0.363	Kashan	0.347	Kerman	0.250
41	Kerman	0.523	Markazi	0.351	Iran	0.232	Dezful	0.245
42	Hormozgan	0.469	Qom	0.319	Shahid-Beheshti	0.229	Markazi	0.239
43	Jiroft	0.443	Dezful	0.245	Tehran	0.224	Zahedan	0.127
44	Zabol	0.015	Yazd	0.161	Markazi	0.215	Yazd	0.033
45	Zahedan	0.009	Rafsanjan	0.104	Yazd	0.215	Rafsanjan	0.000
46	**National average**	**0.756**	**National average**	**0.641**	**National average**	**0.561**	**National average**	**0.563**

 At the outcome level, the average performance score was 0.641 and 62% of the universities scored above the average. Rafsanjan University gained the lowest score (0.104) and the 45^th^ rank, while Shahrud University gained the highest score (0.936) and the first rank among all of the universities (Table 2).

 At the impact level, the average performance score was 0.561 and 37% of the universities gained the scores above average score. Yazd University gained the lowest score (0.215) and the 45^th^ rank, while Qom University gained the highest score (1.00) and the first rank among all of the universities (Table 2).

 As for the composite indicator obtained through the compilation of the indicators of the aforementioned three levels, the average performance score was 0.563 and almost 60% of the universities gained the scores above the average scores. Rafsanjan University gained the lowest score (0.00) and the 45^th^ rank, while Ilam University gained the highest score (1.00) and the first rank among all of the universities (Table 2).

**Figure 1:**
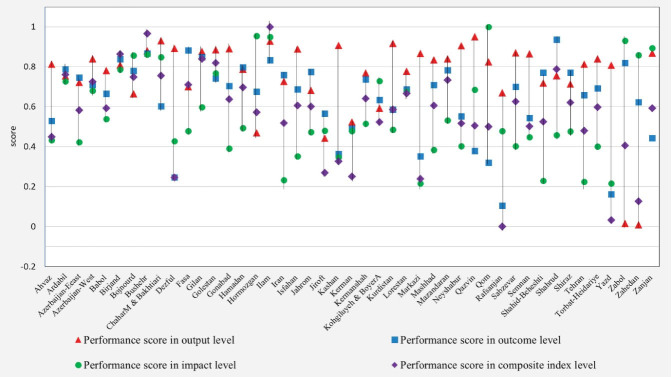
Variations of the Performance Scores of the Medical Sciences Universities in Iran at Four Performance Evaluation Levels.

Figure 1 shows the dispersion and variations of the scores at all four levels. Shorter vertical lines represent smaller variations and greater similarities between the scores obtained at different levels. The performance scores of some universities such as Ardabil, Birjand, Bushehr, and Golestan universities along with the different levels were very similar; hence, with shorter vertical lines. However, the scores of many universities were different; hence, with a longer vertical line. It can be, therefore, concluded that the variations of scores in relation to each other at different levels were considerable and the universities exhibited different behaviors at different evaluation levels.

**Figure 2:**
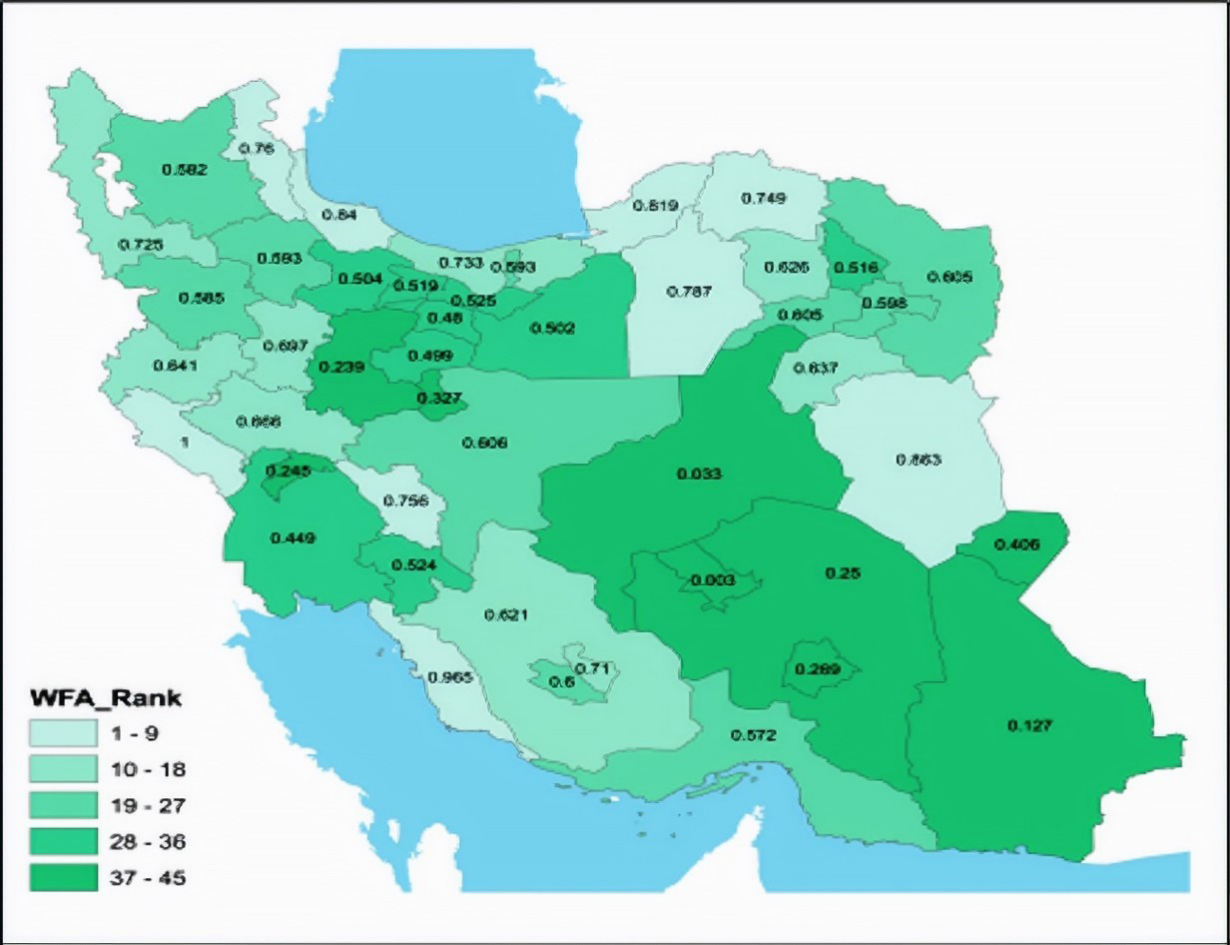
National Map of the Performance Ranks of the Medical Sciences Universities of Iran.

 The national map of the medical sciences universities of Iran is depicted in Figure 2, based on their scores at the composite indicator level (the scores of universities are also shown on the map). Seemingly, those universities with a smaller area under coverage performed better and gained a higher rank compared to the universities with larger areas under coverage.

## Discussion

 The present study was carried out to measure and rank the performance of the medical sciences universities of Iran, regarding improving the health conditions in their target populations. According to the results, the average performance score of the universities on the composite index was 0.563 in 2010. Although 60% of the universities gained the scores higher than the average score, the performance of the universities is considered as unacceptable in general. Therefore, improving the performance of many of these universities is dependent on strict revision of their policies and plans. Since the indicators included in this study reflect the overall health condition in Iran, the resulting scores indicate the average health condition in this country and the necessity of improving the performance for attaining better results in many indicators. Comparing Iran’s health indicators to other countries confirms this statement. In a study by Lim et al, Iran had the 104^th^ rank among 188 countries after gaining a score of 58 on the health-related sustainable development indicators.^35^ Also, a study by Shahraz et al indicated that Iran holds the 13^th^ and 12^th^ ranks among the countries in the region regarding health-adjusted life expectancy and age-standardized mortality rate.^36^ The World Health Organization’s statistics reported that Iran gained the 60^th^, 149^th^, 150^th^, and 153^rd^ ranks among 194 countries regarding the low maternal mortality rate, the under 5 mortality rate, the neonatal mortality rate, and the infants’ mortality rate. As regards the average life expectancy at birth and healthy life expectancy at birth, Iran gained the ranks of 65 and 89, respectively, worldwide.^37^

 The best score on different levels was observed at the output level with an average score of 0.756. This level compromised of several indicators of inclusion in the master plans of the public health deputy, which form the frontline of the services provided by these deputies and are more closely related to the universities’ performances compared to other levels. Therefore, a better condition at this level in comparison with the other levels is not unexpected, and it can be concluded that the management of universities around the globe mainly work on planning and policy-making at this level, therefore gaining satisfactory results because of paying more attention and having specific plans at the coverage indicators level.

 The performance scores of universities on the outcome and impact indicators were 0.641 and 0.561, respectively. Hence, the performance of universities at the outcome level was weaker than their performance at the outcome level; however, it was still better than their performance at the impact level. This level revolves around the disease factors, especially NCDs. The results of this study indicated that universities do not perform well in controlling the risk factors of diseases. Despite the considerable advances in controlling contagious diseases, NCDs are still considered as major health problems in Iran. In the past two decades, the rate of mortality caused by these diseases has increased by 14.5%.^38^ According to the global burden of disease study results in 2017, hypertension, high body mass index, hyperglycemia, tobacco, dietary risks and air pollution accounted for 10.29%, 9.32%, 8.31%, 7.11%, 7.09%, and 5.99%, of the total disease burden in Iran, respectively.^39^ The results of this study reflected the need for national interventions for controlling the risk factors of NCDs in Iran. In the past years, measures such as designing action plans for controlling and preventing NCDs in 2015, setting up a NCDs committee in the Ministry of Health and Medical Education, designing provincial plans for controlling NCDs, and implementing early screening plans for NCDs risk factors and their treatment at low costs (known as the IrPEN program) have been taken to control NCDs in Iran.^40^ However, since these actions affect the final public health outcomes with delay, assessing the effectiveness of these actions calls for continuous monitoring and evaluation of the prevalence of the risk factors and the public health achievements in the Iranian society.

 Also, the lowest mean and the worst condition were observed at the impact level, which revolves around mortality and communicable and NCDs. This level is substantially influenced by the social determinants of health. Besides, public health is not solely affected by the performance of the health system as numerous factors such as a wide range of social and economic determinants affect the public health.Evidently, UMSs cannot control all of these factors and it is not fair to consider these universities as the only entities responsible for the results of the indicators over which they do not have full control. In fact, provision of healthcare services and equitable access to these services are intermediate factors in improving health outcomes, while differences in political, economic, and environmental conditions; social norms; ethnicity; and income can independently or interactively determine the health of a population. Public health is the ultimate outcome of this complicated network of determinants.^36^ The performance score at different levels and their similarity to the real results suggest that the model used in this study performed well in the evaluation of the performances of universities.

 The study results are also indicative of provincial health disparities in Iran. There was considerable dispersion in the performance of the universities at all levels. The scores at the output level varied from 0.950 in Qazvin to 0.009 in Zahedan. The scores at the outcome level varied from 0.936 in Shahrud to 0.104 in Rafsanjan. The scores at the impact level varied from 1.00 in Qom to 0.215 in Yazd, and finally the scores at the composite index level varied from 1.00 in Ilam to 0.00 in Rafsanjan. Other studies have also indicated the disparities in the risk factors and health outcomes in different parts of this country.^15,17,41,42^ For instance, Movahedi et al realized that despite the decrease in disparities of some indicators in different villages of Iran, disparity has been a chronic major problem in the healthcare system of this country in recent years. They also concluded that the pattern of disparities in most indicators is repeated. In that study, the desirability of the indicators in the northern and central provinces and their non-desirability in the eastern and southern provinces of Iran were similar to the present research results, which shows a pattern observed for most indicators.^15^ According to the study by Yazdi and Mahjoob, the rural maternal health indicators in Tehran, Guilan, and Mazandaran provinces were satisfactory. Kohgiluyeh and Boyer-Ahmad and Hormozgan provinces, however, had unsatisfactory indicators, and Sistan and Baluchestan was in a severely unacceptable condition with respect to these indicators.^16^ However, geographical disparities in health outcomes are not solely limited to Iran. For example, Patrick et al performed a study, which revealed that the health outcomes and the related factors were significantly different in different regions of the United States.^27^ The study conducted in Japan also showed that despite the success of this country in reducing the mortalities and morbidities caused by many diseases, the progress has been gradual and regional differences have been growing.^43^ Elimination of these health disparities calls for collaborations among those sectors and organizations that affect the social determinants of health as well as the health sector. Health is an inter-sectoral issue. Besides, the improvement of the health indicators is not solely influenced by the performance of the Ministry of Health and Medical Education. Rather, it is contingent upon collaborations, arrangements, and interactions among all social and economic organizations,^44^ which have been recently stressed by the World Health Organization and many other international organizations.^45^ The integration of the primary healthcare interventions and approaches with the structural and policy changes, which was aimed at improving people’s access to the social determinants of health, is considered as one of the most effective means of reducing disparities in health outcomes.^46^

 In agreement with the present study, different indicator levels were given weights in the United States in a health ranking process.^47,48^ After reviewing the research literature,^49,50^ we selected the PCA method to assign weights to the indicators, whereas in the study carried out in the United States, the expert opinions served this purpose. Also, confirmatory factor analysis (CFA) would be a proper method besides PCA analysis; so, we recommend it for other studies in the same conditions. One of our assumptions was about the unequal effects of different indicators on public health. Hence, the weights of the outcome, output, and impact indicators were not equal in the process of forming the composite indicator and specific weights were assigned to the aforesaid levels through another factor analysis. Seemingly, assigning weights to different levels can increase the likelihood of accepting the research results. In studies conducted in other countries, the same method was used to separate and assign weights to different performance levels, and to identify the final composite performance indicator.^49,50^

 To make optimal use of financial and human resource investments, the public health decisions are expected to be made evidence-informed, which is substantially dependent on the timely provision of accurate and sound information and data. This information is crucial for improving the effectiveness of the decisions made by policy-makers. Besides, it can be used by front-line health providers to improve the quality and efficiency of the health services.^51^ In recent years, numerous national surveys have been conducted in Iran on the health and population. These studies include the Multiple Indicator Health and Demographic Survey and the national surveys of the risks of NCDs. Many attempts have also been made to establish a comprehensive information system in the hospital care sector and the primary healthcare system. However, there is a long road to the development of a comprehensive information system in Iran. The flaws in the health information systems in Iran have challenged the continuous monitoring and performance evaluation of the healthcare organizations. The present study was conducted on the 2010 data due to these information shortages and due to the inaccessibility of information in recent years. Hence, serious measures have to be taken to complete and improve the health information system. Another major limitation related to the lack of access to data was that the development of the target population and its socio-economic level are very influential factors that are not adjusted in the model of this study. Unfortunately, much of the data in the country is produced provincially. So, we had to use provincial data instead of university-based data in some indicators. The situation of social and economic indicators of the general public at the provincial level is almost the same. Given that some of our provinces such as Tehran, Fars, Isfahan, etc. have several universities, it is very difficult to separate the social and economic status of people covered by different universities in a province.

 In Iran, the health system’s plans and policies are generally developed at the national level. The UMSs generally implement the plans and policies developed by the Ministry of Health and Medical Education and despite the local decisions made based on the provincial conditions, many policies are similarly implemented at all universities.^30,52^ The classification of the health achievements of universities using a results chain in this study can effectively guide the regional planners to set the scene for balanced health improvements in different regions of Iran. Our findings also suggest that every UMS can observe its performance at each level compared with others as well as the difference between its scores at different results chain levels to plan improvements in its performance based on the results.

 Furthermore, the public health ranking presented in this study can help direct the current debates to increase awareness, motivation, and dialogue over the solutions with the aim of improving the health outcomes, laying enormous responsibility towards public health, and establishing multilateral collaborations for better outcomes.^27^

 In conclusion, the average performance of universities in Iran at different levels of the results chain is not satisfactory. However, there are considerable differences in the performances of universities. The difference in the performances of some universities at different results chain levels is also considerable. Hence, each university should adopt a strategy suiting its performance level to improve the health conditions. From the output level to the impact level in the results chain, the scores of performance dropped considerably and the impact indicators were in the worst conditions as compared to other levels. Hence, designing effective interventions in proportion to the conditions of universities on different levels of the results chain, developing a robust information system, conducting continuous monitoring and evaluation of public health, performing periodic rankings, organizing competitions over health among universities, and increasing intersectional collaborations are among the fundamental strategies recommended for balanced enhancement of the health outcomes in Iran.

## Supplementary Materials

Supplementary file 1 contains Tables S1-S6.
