# Supplementary material for: Ranking Universities of Medical Sciences as Public Health Services Provider Institutions in Iran: A Result-Chain Analysis
Source: Arch Iran Med. 2022 Apr 1;25(4):214–23. doi: 10.34172/aim.2022.37 (PMC11897872; doi:10.34172/aim.2022.37)
Supplement: Supplementary file 1 — contains Tables S1-S6. [file aim-25-214-s001.pdf]

Supplementary file 1

Table S1. Sources and other details about the indicators used in the study					
**Indicators**	**Type of indicator**	**WHO core indicator**	**Data source**	**Available data/year**	**Data: Uni/Province**
Neonates weighed at the time of birth	output	-	IrMIDHS	2010	province
Percentage of microbiologic quality of drinking water in rural areas	output	-	HPI	2010	university
Percentage of microbiologic quality of drinking water in urban areas	output	-	HPI	2010	university
Accessibility of clean drinking water in rural areas	output	-	HPI	2010	university
Accessibility of sanitary toilets in rural	output	YES	HPI	2010	university
The percentage of consumers of optimal drinking water resources.	output	-	IrMIDHS	2010	province
Sanitary disposal of children's feces	output	-	IrMIDHS	2010	province
Percentage of refined iodized salts in public places and food stores	output	-	HPI	2010	university
The percentage of workshops covered by professional health services	output	-	HPI	2010	university
Percentage of employees covered by occupational examinations	output	-	HPI	2010	university
Prenatal care coverage (at least two times)	output	YES	IrMIDHS	2010	province
Labors carried out in health centers (the public and private sectors)	output	YES	IrMIDHS	2010	province
Prenatal care provided by educated or trained caregivers	output	YES	IrMIDHS	2010	province
The ratio of women who have given birth and have received prenatal supplements.	output	-	IrMIDHS	2010	province
The coverage of users of contraception devices	output	YES	IrMIDHS	2010	province
Children aged between 12 and 23 months, who are vaccinated against measles	output	-	IrMIDHS	2010	province
Prevalence of hypertension	outcome	YES	HPI	2009	province
Prevalence of obesity	outcome	YES	HPI	2009	province
Prevalence of overweight or obesity	outcome	-	HPI	2009	province
Prevalence of hypothyroidism in screened neonates	outcome	-	HPI	2010	university
The rate accurate awareness of HIV prevention in women aged between 15 and 54 years old	outcome	-	IrMIDHS	2010	province
The percentage of children who are exposed to the smoke of cigarettes at least once a week.	outcome	-	IrMIDHS	2010	province
The percentage of children below the age of 5 suffering from diarrhea	outcome	YES	IrMIDHS	2010	province
Prevalence of extreme slimness in children below the age of 5	outcome	YES	IrMIDHS	2010	province
Prevalence of severe dwarfism in children below the age of 5 years	outcome	YES	IrMIDHS	2010	province
The rate of exclusive breastfeeding up to the age of 6 years	outcome	YES	IrMIDHS	2010	province
The prevalence of the birth of underweight neonates	outcome	YES	IrMIDHS	2010	province
The percentage of smokers who smoke daily - men	outcome	YES	NCD	2009	province
The prevalence of the low intake of fruit and vegetable	outcome	-	HPI	2009	province
The prevalence of under-activity	outcome	YES	HPI	2009	province
Successful treatment of new pulmonary tuberculosis cases with positive smears	outcome	YES	HPI	2010	university
Bottle feeding	outcome	-	IrMIDHS	2010	province
Death of neonates below the age of 1 month in every 1000 live births	impact	YES	MPI	2010	province
Death of children below the age of 5 years in every 1000 live births	impact	YES	MPI	2010	province
The still birth to live birth ratio in every 1000 births	impact	-	MPI	2010	province
The prevalence of diabetes in villagers over the age of 30 years	impact	-	HPI	2009	university
The standardized rate of incidence of women's cancers	impact	-	HPI	2009	province
The standardized rate of incidence of men's cancer	impact	-	HPI	2009	province
The total incidence of new tuberculosis cases	impact	YES	HPI	2010	university
Incidence of measles	impact	YES	HPI	2010	province
The incidence of malaria	impact	-	HPI	2010	university

Table S2. Descriptive statistics of indicators

	**Indicators**	**Mean**	**Std. Deviation**		**Median**	**Minimum**	**Maximum**
Output	Neonates weighed at the time of birth	95.16	8.00		97.39	60.22	100.00
	Percentage of microbiologic quality of drinking water in rural areas	87.76	8.57		89.58	68.13	99.38
	Percentage of microbiologic quality of drinking water in urban areas	97.68	3.06		98.89	84.10	100.00
	Accessibility of clean drinking water in rural areas	97.07	4.83		98.47	78.65	100.00
	Accessibility of sanitary toilets in rural	79.40	11.76		80.10	37.40	95.40
	The percentage of consumers of optimal drinking water resources.	96.50	5.21		98.37	77.99	100.00
	Sanitary disposal of children's feces	32.12	11.37		27.30	16.00	62.90
	Percentage of refined iodized salts in public places and food stores	98.48	1.93		99.00	89.20	100.00
	The percentage of workshops covered by professional health services	70.71	18.33		71.00	10.00	95.00
	Percentage of employees covered by occupational examinations	37.60	18.71		35.00	2.00	76.00
	Prenatal care coverage (at least two times)	97.09	2.61		97.19	89.07	100.00
	Labors carried out in health centers (the public and private sectors)	96.03	6.98		98.40	67.58	100.00
	Prenatal care provided by educated or trained caregivers	97.31	2.27		97.31	92.69	100.00
	The ratio of women who have given birth and have received prenatal supplements.	78.68	11.99		81.48	38.05	92.42
	The coverage of users of contraception devices	76.35	6.44		76.84	55.16	85.03
	Children aged between 12 and 23 months, who are vaccinated against measles	96.98	3.78		98.63	85.67	100.00
Outcome	Prevalence of hypertension	147.59	427.10		17.25	10.93	1708.00
	Prevalence of obesity	14.13	3.75		14.21	8.19	22.54
	Prevalence of overweight or obesity	42.38	6.88		43.14	25.19	55.79
	Prevalence of hypothyroidism in screened neonates	245.76	120.27		211.00	52.00	636.00
	The rate accurate awareness of HIV prevention in women aged between 15 and 54 years old	31.23	7.09		32.46	13.84	43.33
	The percentage of children who are exposed to the smoke of cigarettes at least once a week.	19.41	7.78		20.11	4.49	47.90
	The percentage of children below the age of 5 suffering from diarrhea	14.76	5.68		14.76	5.46	23.47
	Prevalence of extreme slimness in children below the age of 5	1.34	1.06		1.17	0.00	5.05
	Prevalence of severe dwarfism in children below the age of 5 years	1.94	1.50		1.56	0.00	5.97
	The rate of exclusive breastfeeding up to the age of 6 years	54.15	11.67		55.32	33.11	85.65
	The prevalence of the birth of underweight neonates	4.12	3.15		2.96	0.56	12.88
	The percentage of smokers who smoke daily - men	19.05	4.66		18.38	10.78	28.42
	The prevalence of the low intake of fruit and vegetable	88.70	6.35		89.69	68.21	99.50
	The prevalence of under-activity	38.60	8.86		38.44	20.42	53.87
	Successful treatment of new pulmonary tuberculosis cases with positive smears	80.04	10.53		80.00	50.00	100.00
	Bottle feeding	30.18	6.25		31.56	14.57	40.12
Impact	Death of neonates below the age of 1 month in every 1000 live births	10.21	3.72		10.82	3.22	18.52
	Death of children below the age of 5 years in every 1000 live births	16.93	5.36		17.82	5.86	29.77
	The still birth to live birth ratio in every 1000 births	8.05	4.01		7.42	0.45	15.29
	The prevalence of diabetes in villagers over the age of 30 years	4.17	1.77		3.54	1.96	9.57
	The standardized rate of incidence of women's cancers	113.76	35.45		126.03	32.83	172.74
	The standardized rate of incidence of men's cancer	122.87	34.26		131.61	35.99	179.90
	The total incidence of new tuberculosis cases	16.49	19.59		11.40	3.70	104.00
	Incidence of measles	7.68	14.07		4.00	0.00	68.00
	The incidence of malaria	3.36	14.31		0.20	0.00	93.60

**Table S3.** Different steps of assigning weights to the indicators of output level						
**Indicators**	**Squared Factor Loading Scaled to sum to 1**		**The largest weight of indicators**	**Weight of the Respective Factor**	**Weight Score(Wi)**	**Resulting Weight(ΣWi=1)**
	**Factor 1**	**Factor 2**				
Neonates weighed at the time of birth	0.140	0.002	0.140	0.627	0.088	0.145
Percentage of microbiologic quality of drinking water in rural areas	0.003	0.307	0.307	0.132	0.040	0.067
Percentage of microbiologic quality of drinking water in urban areas	0.061	0.005	0.061	0.627	0.038	0.063
Accessibility of clean drinking water in rural areas	0.027	0.066	0.066	0.132	0.008	0.0145
The percentage of consumers of optimal drinking water resources.	0.121	0.000	0.121	0.627	0.076	0.126
Sanitary disposal of children's feces	0.015	0.002	0.015	0.627	0.009	0.016
Percentage of refined iodized salts in public places and food stores	0.008	0.000	0.008	0.627	0.005	0.008
The percentage of workshops covered by professional health services	0.051	0.003	0.051	0.627	0.032	0.053
Percentage of employees covered by occupational examinations	0.036	0.051	0.051	0.132	0.006	0.011
Prenatal care coverage (at least two times)	0.110	0.135	0.135	0.132	0.017	0.029
Labors carried out in health centers (the public and private sectors)	0.138	0.001	0.138	0.627	0.086	0.143
Prenatal care provided by educated or trained caregivers	0.062	0.235	0.245	0.132	0.032	0.053
The ratio of women who have given birth and have received prenatal supplements.	0.024	0.000	0.024	0.627	0.015	0.025
The coverage of users of contraception devices	0.104	0.000	0.104	0.627	0.065	0.108
Children aged between 12 and 23 months, who are vaccinated against measles	0.090	0.005	0.090	0.627	0.057	0.094
Accessibility of sanitary toilets in rural	0.000	0.169	0.169	0.132	0.022	0.037
Sum	1	1	-	-	0.605	1
Variance ratio described by the related factor	0.627	0.132	-	-	-	-

**Table S4.** Different steps of assigning weights to the indicators of outcome level							
**Indicators**	**Squared Factor Loading Scaled to sum to 1**			**The largest weight of indicators**	**Weight of the Respective Factor**	**Weight Score(Wi)**	**Resulting Weight(ΣWi=1)**
	**Factor 1**	**Factor 2**	**Factor 2**				
Prevalence of hypertension	0.38	0.083	0.041	0.083	0.147	0.012	0.021
Prevalence of obesity	0.142	0.017	0.000	0.142	0.434	0.061	0.108
Prevalence of overweight or obesity	0.192	0.000	0.000	0.192	0.434	0.083	0.147
Prevalence of hypothyroidism in screened neonates	0.004	0.064	0.43	0.064	0.147	0.009	0.016
The rate accurate awareness of HIV prevention in women aged between 15 and 54 years old	0.039	0.070	0.063	0.070	0.147	0.010	0.018
The percentage of children who are exposed to the smoke of cigarettes at least once a week.	0.001	0.001	0.239	0.239	0.147	0.035	0.062
The percentage of children below the age of 5 suffering from diarrhea	0.010	0.008	0.286	0.289	0.147	0.042	0.074
Prevalence of extreme slimness in children below the age of 5	0.90	0.171	0.001	0.171	0.147	0.025	0.044
Prevalence of severe dwarfism in children below the age of 5 years	0.171	0.003	0.004	0.171	0.434	0.074	0.0131

The rate of exclusive breastfeeding up to the age of 6 years	0.010	0.158	0.018	0.158	0.147	0.023	0.041
The prevalence of the birth of underweight neonates	0.199	0.000	0.000	0.199	0.434	0.086	0.153
The percentage of smokers who smoke daily - men	0.049	0.071	0.000	0.071	0.147	0.010	0.018
The prevalence of the low intake of fruit and vegetable	0.033	0.151	0.009	0.151	0.147	0.022	0.039
The prevalence of under-activity	0.020	0.180	0.007	0.180	0.147	0.026	0.047
Successful treatment of new pulmonary tuberculosis cases with positive smears	0.016	0.015	0.032	0.032	0.147	0.004	0.008
Bottle feeding	0.000	0.000	0.249	0.249	0.147	0.036	0.065
Sum	1	1	1	-	-	0.567	1
Variance ratio described by the related factor	0.434	0.147	0.147	-	-	-	-

Table S5. Different steps of assigning weights to the indicators of impact level

**Indicators**	**Squared Factor Loading Scaled to sum to 1**			**The largest weight of indicators**	**Weight of the Respective Factor**	**Weight Score(Wi)**	**Resulting Weight(ΣWi=1)**
	**Factor 1**	**Factor 2**	**Factor 3**				
Neonatal mortality rate(per thousand live births)	0.399	0.000	0.011	0.399	0.372	0.148	0.178
Under 5 mortality rate(per thousand live births)	0.401	0.000	0.004	0.401	0.372	0.149	0.179
The rate of stillbirth per 1000 births	0.130	0.053	0.024	0.130	0.372	0.048	0.058
The prevalence of diabetes in people over 30 years old in rural areas	0.032	0.058	0.001	0.058	0.357	0.021	0.025
The standardized incidence rate of women's cancer	0.007	0.400	0.018	0.400	0.357	0.143	0.171
The standardized incidence rate of men's cancer	0.001	0.345	0.060	0.345	0.357	0.123	0.148
The incidence of TB(positive smear)	0.029	0.001	0.178	0.178	0.226	0.040	0.048
The incidence of measles	0.000	0.081	0.463	0.463	0.226	0.105	0.125
The incidence of malaria	0.000	0.062	0.242	0.242	0.226	0.055	0.066
Sum	1.000	1.000	1.000	-	-	0.831	1.00
Variance ratio described by the related factor	0.372	0.357	0.226	-	-	-	-

Table S6. Different steps of assigning weights to the composite index

**Indicators**	**Squared Factor Loading Scaled to sum to 1**		**The largest weight of indicators**	**Weight of the Respective Factor**	**Weight Score(Wi)**	**Resulting Weight(ΣWi=1)**
	**Factor 1**	**Factor 2**				
output indicators score	0.594	0.019	0.594	0.435	0.259	0.334

outcome indicators score	0.000	0.944	0.944	0.359	0.338	0.437
impact indicators score	0.404	0.036	0.404	0.435	0.176	0.227
Sum	1	1	-	-	0.774	1
Variance ratio described by the related factor	0.435	0.359	-	--	-	-
